# Fusion of Antigen to a Dendritic Cell Targeting Chemokine Combined with Adjuvant Yields a Malaria DNA Vaccine with Enhanced Protective Capabilities

**DOI:** 10.1371/journal.pone.0090413

**Published:** 2014-03-05

**Authors:** Kun Luo, Hong Zhang, Fidel Zavala, Arya Biragyn, Diego A. Espinosa, Richard B. Markham

**Affiliations:** 1 The Department of Molecular Microbiology and Immunology, Johns Hopkins Bloomberg School of Public Health, Baltimore, Maryland, United States of America; 2 Immunoregulation Section, Laboratory of Molecular Biology and Immunology, National Institute on Aging, Baltimore, Maryland, United States of America; Tulane University, United States of America

## Abstract

Although sterilizing immunity to malaria can be elicited by irradiated sporozoite vaccination, no clinically practical subunit vaccine has been shown to be capable of preventing the approximately 600,000 annual deaths attributed to this infection. DNA vaccines offer several potential advantages for a disease that primarily affects the developing world, but new approaches are needed to improve the immunogenicity of these vaccines. By using a novel, lipid-based adjuvant, Vaxfectin, to attract immune cells to the immunization site, in combination with an antigen-chemokine DNA construct designed to target antigen to immature dendritic cells, we elicited a humoral immune response that provided sterilizing immunity to malaria challenge in a mouse model system. The chemokine, MIP3αCCL20, did not significantly enhance the cellular infiltrate or levels of cytokine or chemokine expression at the immunization site but acted with Vaxfectin to reduce liver stage malaria infection by orders of magnitude compared to vaccine constructs lacking the chemokine component. The levels of protection achieved were equivalent to those observed with irradiated sporozoites, a candidate vaccine undergoing development for further large scale clinical trial. Only vaccination with the combined regimen of adjuvant and chemokine provided 80–100% protection against the development of bloodstream infection. Treating the immunization process as requiring the independent steps of 1) attracting antigen-presenting cells to the site of immunization and 2) specifically directing vaccine antigen to the immature dendritic cells that initiate the adaptive immune response may provide a rational strategy for the development of a clinically applicable malaria DNA vaccine.

## Introduction

The 1967 report by Nussenzweig et al [1] that immunization with irradiated sporozoites could induce sterilizing immunity to malaria in mice has provided the foundation for current efforts at malaria vaccine development. A recent clinical trial of this approach demonstrated the need for five rounds of intravenous immunizations in order to uniformly attain sterilizing immunity [2] Intensive study of the immune mechanisms mediating efficacy of irradiated sporozoite vaccines has led to a focus on the important role of liver-resident CD8 T cells in the development of the observed protection [3]–[10].

Because of the impracticality of generating and administering the large quantities of irradiated sporozoites that would be needed for protection within malaria endemic areas, efforts to develop subunit vaccines have persisted. The recently completed, and most extensive to date, clinical trial of a subunit vaccine capable of eliciting both humoral and T-cell mediated immune responses demonstrated only 35% protection against the development of severe malaria [11].

Given the paucity of examples of clinically successful vaccines acting through cell-mediated immune mechanisms, it becomes important to acknowledge the obstacles that confront the development of any vaccine targeting that mechanism of protection. Elicitation of effective T-cell mediated protective immunity targeting liver stage parasites may be problematic because of evidence that 1) persistent antigen exposure is most effective at eliciting effector T cell memory [3], [12] and 2) exposure to malaria sporozoites gives rise to a short burst of CD8 T cell responses that are refractory to re-stimulation [13], [14]. Further, critical to T-cell mediated protection is the necessity for sustaining local memory T cells at the site of parasite invasion, in this case, the liver. A recent study with a candidate herpes virus type 2 vaccine has demonstrated that localized application of chemokine preparations to the potential site of virus exposure can ensure local maintenance of effector memory T cells and enhanced resistance to infection within the female genital tract of mice [15]. It is unclear how such localization and maintenance of memory T cells could be obtained within the liver. Indeed, the prodigious, but unsuccessful, effort to develop a T cell vaccine to prevent human immunodeficiency virus infection, culminating in the large STEP study [16] provides ample testimony to the difficulty of achieving this goal.

In view of these experiences, our laboratory has undertaken an effort to develop a malaria vaccine that can provide protective immunity simply on the basis of its ability to elicit a profound humoral immune response. That humoral immunity can protect against infection initiated with as many as 100 sporozoites inoculated intravenously has been demonstrated with passive administration of both monoclonal and polyclonal antibodies, as well as with immunization with adjuvanted peptide vaccines [17]–[20]. However, elicitation of equivalent protective humoral immunity with DNA vaccines has been difficult to achieve (reviewed in [21].

For these studies we employ a mouse strain, C57Bl/6 (H-2b) that poses a high bar to the development of protective immunity. C57Bl/6 mice are known to lack T cells within their repertoire capable of generating a Class I-restricted T cell response to the *Plasmodium yoelii* malaria circumsporozoite protein [22], [23]. Further, in studies with *Plasmodium berghei* this mouse strain is more susceptible to infection than the Balb/c strain often used in malaria vaccine studies [24], [25], rendering the demonstration of protective immunity more difficult. C57Bl/6 mice have been identified, therefore, as the preferred mouse strain for use in studies of immune protection against *P. berghei* liver stage infection [25] and in the current studies we will be analyzing the ability of these mice to be protected against parasite challenge that is an order of magnitude greater than that used in the previously cited humoral immunity studies.

The immunodominance of the circumsporozoite protein in sporozoite-elicited immunity to malaria has been well-described [22]. The use of adjuvants to enhance the immune response represents an effort to attract to the site of immunization those cells that initiate the development of acquired immunity, without eliciting the adverse inflammatory effects associated with true infection. However, many of the cells attracted by adjuvants play no direct role in the development of an immune response [26]. As part of the effort to attract cells involved in the initiation of the immune response without producing excessive inflammation, considerable interest has developed in using specific chemokines to replace the more non-specifically acting group of adjuvants based primarily on bacterial lipid derived products [27]–[29]. To enhance the efficiency of bringing the antigen into contact with appropriate antigen presenting cells, several studies have examined generating vaccines by fusing candidate vaccine antigens to chemokines that attract the appropriate antigen presenting cells to the site of immunization [28], [30], [31]. While many of these studies have focused on the ability of specific cytokines or chemokines to enhance the inflammatory infiltrate or promote differentiation of specific subsets of immune cells [27], [28], [32]–[34], others have used chemokines to target pathogen proteins to specific immune cell populations. In many of these studies, the chemokine was expected to perform the dual function of attracting cells to the site of immunization and then targeting the pathogen-specific antigen to an immune cell of interest, frequently antigen-presenting dendritic cells [35]–[40].

In the current study we have examined the merit of treating these two functions of adjuvants, chemoattractant and cell-targeting, as independent variables in the development of a DNA vaccine. This approach allowed us to develop vaccine strategies that optimize both variables independently. To stimulate the innate immune system, we employed the adjuvant Vaxfectin, which has been demonstrated to function well as an adjuvant for DNA vaccines and has specifically been shown to enhance the response to candidate malaria DNA vaccines [41]–[44]. Because the initiation of the immune response requires efficient engagement of antigen with immature dendritic cells, we fused DNA encoding portions of the circumsporozoite protein (CSP) from either the mouse malaria strain *Plasmodium yoelii* or the human malaria strain *Plasmodium falciparum* to DNA encoding the chemokine macrophage inflammatory protein-3α(MIP3α), also known as CCL20.

Our studies indicate that the addition of DNA encoding MIP3α to the CSP DNA construct provided no significant alteration of the cellular or cytokine or chemokine profile obtained at the immunization site over that achieved with Vaxfectin and CSP DNA alone. As used in this vaccine construct, the MIP3α component lacked any of the traditional adjuvant functions. However, the presence of the MIP3α component in the vaccine DNA construct, when combined with the use of Vaxfectin, resulted in profound enhancement in mice of the antibody response and levels of protection against malaria sporozoite challenge equivalent to those observed after immunization with irradiated sporozoites, the gold standard in the field. Vaccine design which incorporates the independent roles of the chemoattractant and cell-targeting components in eliciting immune responses can lead to dramatic improvements in the humoral immune response and the resulting vaccine efficacy.

## Materials and Methods

### Animals

Six- to eight-week-old female C57BL/6 (H-2b) mice were purchased from The Jackson Laboratory (Bar Harbor, ME) and maintained in a pathogen-free micro-isolation facility in accordance with the National Institutes of Health guidelines for the humane use of laboratory animals. All experimental procedures involving mice were approved by the Institutional Animal Care and Use Committee of the Johns Hopkins University (Protocol Number MO6H319).

### Plasmids

**Figure 1:**
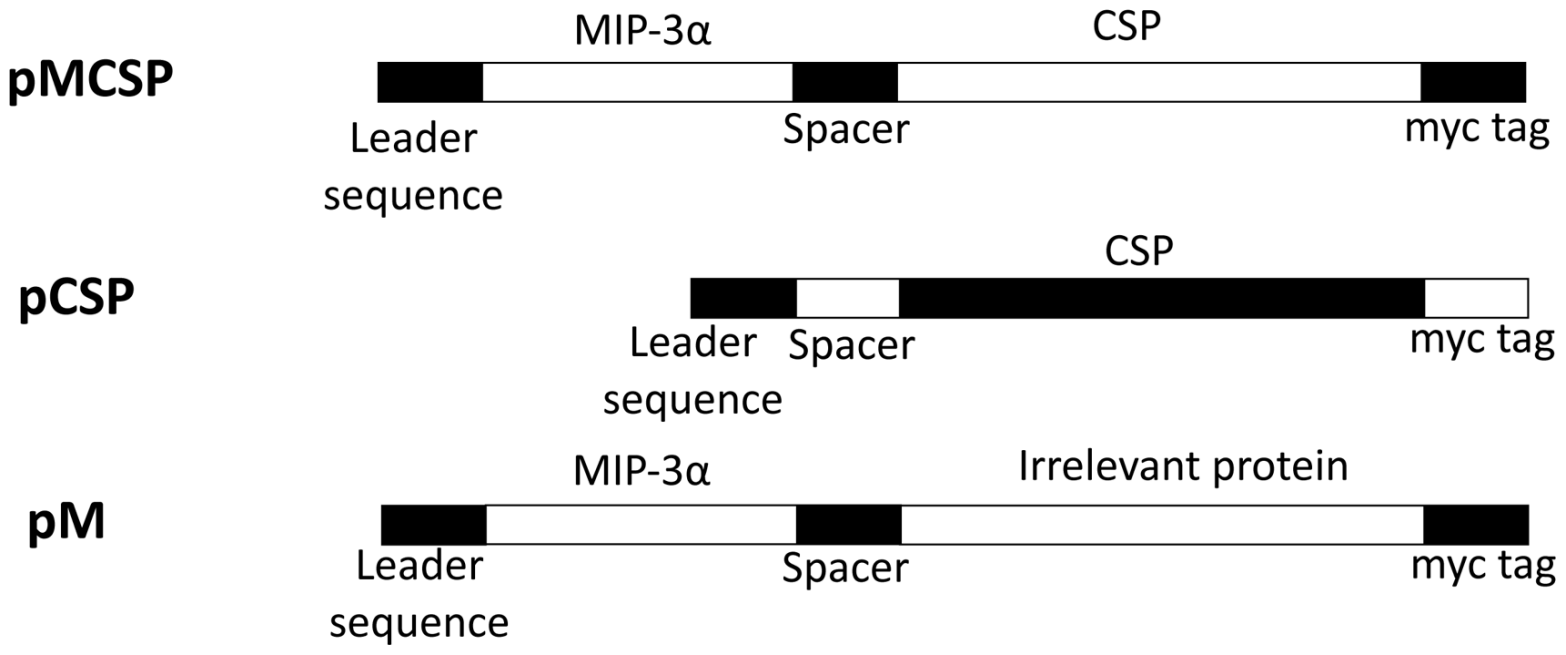
Diagrammatic representation of the DNA constructs, including control plasmids, used for immunization. The construct for *P. yoelii* used mouse MIP3α, while the *P. falciparum* construct employed the sequence for human MIP3α. M refers to MIP3α. CSP refers to a segment of the *Plasmodium yoelii* circumsporozoite protein; and MCSP refers to the fusion protein of MIP-3α and a segment of the *P*. *yoelii* circumsporozoite protein. Also shown are the positions of the leader sequence, spacer, and the myc tag. The leader sequence for the *P. yoelii* construct is the interferon–γ induced protein 10 secretion leader sequence; the leader sequence for the *P. falciparum* construct is the human tissue plasminogen activator leader sequence [46]. The MCSP and hMfCSP nucleotide sequences are provided in the supplementary materials.

The plasmid DNA encoding regions of the *P. yoelii* or *P. falciparum* circumsporozoite protein (pCSP) fused with murine or human MIP-3α (pMCSP) and control plasmids are described in Figure 1. Plasmid (pM) containing DNA encoding MIP-3α fused to DNA encoding an irrelevant lymphoma immunoglobulin variable region protein [39] was used as a negative control. The leader sequence for the *P. yoelii* construct is the interferon-γ-induced protein 10 (IP-10) leader sequence. The *P*. *yoelii* and *P. falciparum* circumsporozoite nucleotide and amino acid sequences are provided in the supplementary materials. For construction of the *P. falciparum* DNA vaccine, plasmid (phMfCSP) encoding human MIP-3α fused with a codon-optimized *P. falciparum* (3D7) CSP DNA sequence containing deletions of the N-terminal 20 aa signaling sequence and the C-terminal 23 aa anchor region was synthesized and cloned into the DNA vaccine plasmid VR1012 [45]. Human MIP3α was employed in this construct to validate its efficacy since it would be the component employed in any human vaccine targeting *P. falciparum*. Both mouse and human MIP3α can serve as ligands for mouse CCR6. The human tissue plasminogen activator (TPA) signal sequence [46] was included in the synthetic construct of hMfCSP and fCSP to obtain more efficient protein secretion than that observed with the *P. falciparum* construct using the IP-10 leader sequence (data not shown). Control plasmids (pfCSP and phM) were constructed using the same strategies as the constructs pCSP and pM. Plasmids were purified using Endofree purification columns (Qiagen, Hilden, Germany) and stored at -20° C in PBS. Endotoxin contaminates were determined using the ToxinSensorTM Chromogenic LAL Endotoxin Assay Kit (Genscript, NJ). All plasmids used for immunization had final endotoxin levels below, the lower limit of detection of the kit, 0.1 EU/DNA. The DNA sequences for the leader, MIP3α spacer and CSP components of the vaccine plasmids are provided in Figures S1 and S2.

### Transfection and Western Blot

DNA transfection in 293T cells was carried out with Lipofectamine 2000 (Invitrogen, Carlsbad, CA) as described by the manufacturer. Cells and supernatant were collected 48 h after transfection. Lysed cells (10^5^) and supernatant (10µl) were placed in loading buffer (0.08 M Tris (pH 6.8), 2.0% sodium dodecyl sulfate (SDS), 10% glycerol, 0.1 M dithiothreitol, 0.2% bromophenol blue). The samples were boiled for 10 min, and proteins were separated by SDS-polyacrylamide gel electrophoresis. Proteins were transferred onto nitrocellulose membranes, and the membranes were probed with anti-cMyc antibodies, kindly provided by Dr. Michael Matunis, Johns Hopkins Bloomberg School of Public Health, Baltimore, MD. Secondary antibodies were alkaline phosphatase-conjugated anti-mouse antibodies (Jackson Immunoresearch, Inc., West Grove, PA), and staining was carried out with 5-bromo-4-chloro-3-indolylphosphate (BCIP) and nitro blue tetrazolium (NBT) solutions prepared from chemicals obtained from Sigma (St. Louis, MO).

### Vaxfectin formulation

The adjuvant Vaxfectin has been previously described [47] and was provided by Vical Inc. (San Diego, CA). Vaccine formulations were prepared by adding 1 ml of 0.9% NaCl solution to 2.18 mg of Vaxfectin, which was then mixed in an equivalent volume with 2 mg/ml DNA. The mixture was then diluted to the desired DNA concentration with PBS. The final DNA/Vaxfectin molar ratio was 4∶1.

### Immunization of mice

C57BL/6 mice were immunized with 2 µg (*P. yoelii*) or 5 µg (*P. falciparum*) of the constructs described above, and were delivered as a single intramuscular injection in the anterior tibialis muscle in 100µl of PBS formulated with Vaxfectin. Mice received three immunizations at bi-weekly intervals. For the positive control group for the *P. yoelii* studies, 10^5^ (initial immunization) and 5×10^4^ (booster immunizations) with irradiated *P. yoelii* sporozoites (17XN) obtained from *Anopheles stephensi* mosquitoes maintained in the Johns Hopkins Malaria Research Institute insectary were inoculated by tail-vein injection at the same time-points.

### Parasites for challenge

*P. yoelii* and transgenic *P. berghei* sporozoites [48] were used for challenge. Sporozoites were obtained by hand dissection of salivary glands of *Anopheles stephensi* mosquitoes maintained in the Johns Hopkins Malaria Research Institute insectary. The isolated sporozoites were suspended in HBSS medium containing 1% normal mouse serum. Challenges to evaluate vaccine effect on hepatic parasite load were accomplished by injecting 5×10^3^ sporozoites in the tail vein. Challenges to evaluate protection from blood stage malaria were accomplished by injecting 1×10^3^ sporozoites in the tail vein.

### ELISA assay

Humoral immune responses to the immunodominant B cell epitope of the relevant CSP protein were measured using a previously described CSP-specific ELISA, with minor modifications [49]. ELISA plates were coated with 2 µg of recombinant PyCSP or recombinant PfCSP. Recombinant PyCSP was a GST-tagged segment of PyCSP protein that includes 15 copies of the central GPGAPQ repeat region [50]. Recombinant PfCSP was a 6-His-tagged segment of the PfCSP protein that includes 21 copies of the central NANP repeat region and was kindly provided by Dr. Gary Ketner, Johns Hopkins Bloomberg School of Public Health, Baltimore, MD. The endpoint ELISA titer is reported as the highest dilution of serum at which the average absorbance was twice the value obtained using pre-immunization serum. For quantitation of the antibody concentration, monoclonal 2A10 [51], [52] (IgG2b, κ), which recognizes the (NANP)_3_ sequence of *P. falciparum* was used as the standard. Known concentrations of this standard were diluted to obtain O.D._405_ readings in the linear range and compared to equivalent O.D._405_ readings with dilutions of sera from immunized mice. For statistical analysis of differences in antibody concentrations among different groups, direct O.D._405_ readings of individual mice were used.

To obtain ELISA readings, plates were placed inside a humidity chamber and incubated overnight at 4°C. Plates were blocked with 2% BSA for 30 min at room temperature (RT) and then serially diluted serum samples were added and incubated at RT for 2 h. After washing six times, peroxidase labeled goat anti-mouse IgG (Jackson ImmunoResearch Laboratories, INC) was added at a dilution of 1∶1,000 and incubated at RT for 1 h. After washing six times, ABTS Peroxidase substrate (KPL, Gaithersburg, MD) was added for development and incubated for 1 h. The data were collected using the Synergy HT (BioTek Instruments, Inc, Winooski, VT).

### Real-time PCR for liver stage parasites

Real-time PCR was used for the detection and quantification of the liver stage of Plasmodium parasites [53]. Two pairs of specific primers were designed to amplify the parasite 18s rRNA sequence. For *P. yoelii* parasites (17XNL), the forward primer was 5′-GGGGATTGGTTTTGACGTTTTTGCG-3′ and the reverse primer was 5′-AAGCATTAAATAAAGCGAATACATCCTTAT-3′. For transgenic *P.berghei* parasites, the forward primer was 5′-TGGGAGATTGGTTTTGACGTTTATGT-3′ and the reverse primer was 5′-AAGCATTAAATAAAGCGAATACATCCTTAC-3′. Values were normalized against measurements of mouse actin mRNA in the same samples. The primers used for mouse actin were as follows: 5′-GTCCCTCACCCTCCCAAAAG-3′ (forward) and 5′-GCTGCCTCAACACCTCAACCC-3′ (reverse). The reactions were performed in a final volume of 20 µl using SYBR green PCR Master Mix (2X) from Applied Biosystems and processed with ABI StepOne Real-time PCR system (Applied Biosystems). Amplification was performed using a 2-min step at 50°C and a 10-min denaturation step at 95°C, followed by 40 cycles of 15 s denaturation at 95°C, 1 min of primer annealing, and polymerization at 60°C.

### In vivo depletion of T cell subsets

To deplete the CD4+, CD8+, or both T cell subsets, immunized mice were injected i.p. with anti-CD4 (GK1.5) and/or anti-CD8 (2.4.3) or both mAbs, purchased from American Type Culture Collection (ATCC, Rockville, MD]. Each mouse received daily doses of 200 µg of anti-CD4 or anti-CD8 or both antibodies for two days. Twenty-four hours after the last mAb treatment, the efficacy of the depletion was estimated by two-color flow cytometry analysis of peripheral blood lymphocytes, using FITC-conjugated anti-mouse CD4 or APC-conjugated anti-CD8 mAbs (eBioscience,San Diego, CA).

### Real-time PCR analysis of cytokine and chemokine levels of muscle samples

Total RNA was isolated from tissue samples (n = 3 per group). Reverse transcription was done using total RNA (2 µg) as template and the High-Capacity cDNA reverse transcription kit (Applied Biosystems; Foster City, CA) reagents. Relative transcript levels for CXCL-2, IFN-γ, CXCL-9, TGF-β, CCL-2, TNF-α, IL-2, IL-6, IL-10, IL-12b, and MIP-3α were determined in tissue samples collected 24 h and 48 h after vaccination. For this purpose commercially available TaqMan Gene Expression Assays (Applied Biosystems) were used. These cytokines and chemokines were selected for analysis based on preliminary exploratory work on biomarkers associated with Vaxfectin formulations [54]. Analysis was performed using the ABI Prism 7900HT Sequence Detection System (Applied Biosystems) and cycling conditions suggested by the manufacturer. A comparative method was used to determine relative quantitation. Briefly, Ct values for target gene amplification were normalized by subtracting the Ct values for the house keeping gene GAPDH.

### Extraction and analysis of infiltrating DCs

Anterior tibialis muscles from immunized mice were harvested after euthanization at the indicated time points, minced, and incubated with digestion buffer (Hanks' Balanced Salt Solution (Invitrogen, Carlsbad, CA), supplemented with 0.5% collagenase, 0.2% BSA, and 0.025% trypsin) for 30 minutes at 37°C with vortexing every 5 minutes to facilitate tissue dissociation. Cell suspensions were filtered, washed twice with PBS containing 2% FBS. The cells numbers were counted and 1×10^6^ were stained with APC-labeled CD11c antibodies (clone N418, eBioscience, San Diego, CA), and analyzed by flow cytometry on a Becton-Dickinson FACScan (Becton-Dickinson, Franklin Lakes, New Jersey).

### Statistical analysis

Differences in sporozoite rRNA and antibody concentrations among groups were analyzed by an ANOVA test (Stata Corp, College Station, TX). A value of *p*<0.05 was considered to be significant. Correlations between antibody concentration and *P. yoelii* rRNA levels in the liver were determined using Pearson's r correlation (pwcorr) function (Stata Corp, College Station, TX). The significance of differences in development of bloodstream infection among groups of challenged mice was determined using a Fisher exact test (Stata Corp, College Station, TX).

## Results

### Expression of vaccine constructs

**Figure 2:**
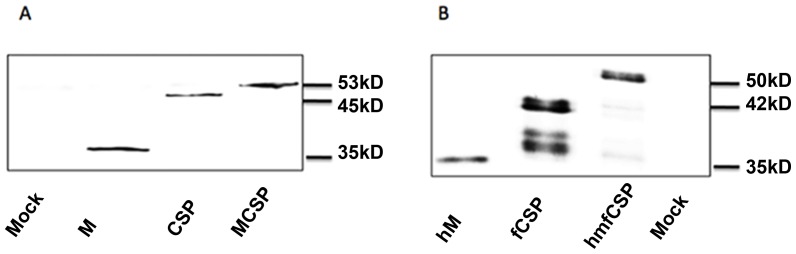
Western blot analysis of expression of constructs of malaria DNA vaccines and controls. *P. yoelii* (A) and *P. falciparum* (B) DNA vaccine candidates and control constructs were transfected into and expressed from 293T cells. Protein expression into culture supernatant was detected with anti-myc antibody 48 hours after transfection. In each lane the vaccine constructs are the uppermost bands with lower bands of fCSP representing proteolytic fragments of the expressed proteins. CSP = a fragment of the *P. yoelii* circumsporozoite protein, fCSP = a fragment of the *P. falciparum* circumsporozoite protein, M = murine MIP3α, hM = human MIP3α.

The efficacy of any DNA vaccine targeting the elicitation of a humoral immune response and dependent on the activity of incorporated targeting ligands will depend on achieving extracellular expression of the encoded proteins. Figure 2 demonstrates that both the *P. yoelii* and *P. falciparum* constructs could be expressed into the extracellular environment from transformed 293T cells. In order to achieve efficient expression of the *P. falciparum* construct, the human tissue plasminogen activator leader sequence was used. Both constructs with and without the MIP3α component were expressed as soluble proteins in the extracellular environment. The fCSP lane shows the presence of proteolytic fragments, as has been observed by others expressing this construct [55].

### Comparison of immunization regimens for elicitation of antibody responses

**Figure 3:**
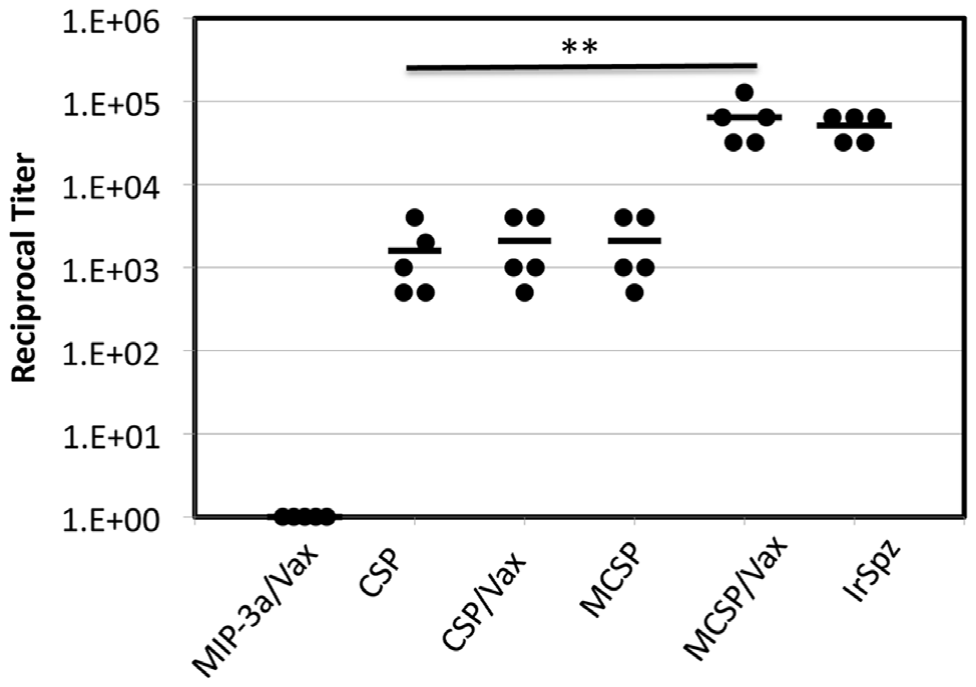
Antibody titers in C57Bl/6 mice immunized with *P. yoelii* CSP DNA constructs Specific antibody concentrations in sera obtained two weeks after the final immunization. Values shown represent the reciprocal of the endpoint ELISA titer for each mouse (closed circles) and the mean of the reciprocal titers from the 5 mice in each group (horizontal broken lines). The endpoint titer is reported as the highest dilution of serum at which the absorbance was twice the value obtained using pre-immunization serum. The responses to irradiated sporozoite and MIP3α-CSP/Vaxfectin immunization regimens did not differ (p>0.9) and were significantly greater than those to the other immunization regimens (p<0.001), as indicated by ** in the figure.

To test the relative immunogenicity of the different *P. yoelii* vaccine constructs with and without Vaxfectin, C57BL/6 mice were immunized three times with a low dose (2 µg) of the different vaccine or control plasmids according to the schedule outlined in the Methods. For the positive control group (IrSpz), 10^5^ (initial immunization) and 5×10^4^ (booster immunizations) irradiated *P. yoelii* sporozoites (17XN) were inoculated at the same time-points. Mice were bled two weeks after the final immunization to determine specific antibody concentrations. Figure 3 demonstrates the differences in endpoint dilution titers from individual mice and the mean endpoint dilution titer for each group. The results indicate that fusion of DNA encoding MIP3α to the CSP DNA vaccine construct or use of the CSP DNA vaccine construct in combination with Vaxfectin both resulted in similar enhancement of the antibody response which, in this experiment, was marginally, but not significantly improved over the response to CSP alone (p = 1.000). However the response to the combination of Vaxfectin plus the MIP3α-CSP DNA construct was significantly greater than responses to the other CSP-containing vaccine regimens (p<0.001) and did not differ significantly from the response to irradiated sporozoites (p>0.9).

### Protective efficacy of different vaccine regimens to sporozoite challenge

**Figure 4:**
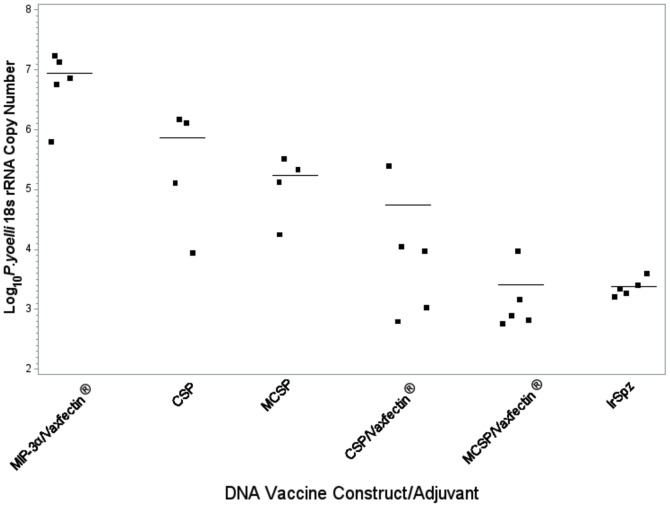
Effect of immunization with the combination of Vaxfectin and MCSP DNA on protection achieved against *in vivo* challenge with *P. yoelii* sporozoites. C57BL/6 mice were immunized with the indicated DNA vaccine or irradiated sporozoites as described in the Methods section. Two weeks after the final immunization, mice were challenged with 5×10^3^ *P. yoelii* sporozoites, and parasite-specific rRNA levels in the liver were determined by quantitative RT-PCR on samples obtained 48 hours post challenge. All results were normalized against the expression of actin. Results indicate the copy numbers from individual mice (closed squares) and the mean from the 5 mice in each group (horizontal lines). The MCSP/Vaxfectin or irradiated sporozoite groups differed significantly from any other group (p<0.002), but not from each other (p>1.000).

To determine if the differences in antibody responses among the different regimens correlated with protection, the immunized mice were challenged two weeks after the final immunization with 5×10^3^ *P. yoelii* sporozoites obtained by dissection of the salivary glands of infected *A. stephensi* mosquitoes. Liver sections recovered 48 hours post-challenge from five mice from each of the immunization groups were assayed by qRT-PCR for the level of *P. yoelii* 18s rRNA present (Figure 4). Compared to mice immunized with a construct lacking CSP, mice immunized with CSP alone had an approximately one log reduction in the *P. yoelii* rRNA load in the liver, while recipients of either MCSP alone or CSP plus Vaxfectin had their parasite load reduced 100 fold. Recipients of the MCSP plus Vaxfectin or irradiated sporozoites vaccines had an essentially equivalent 3.5 order of magnitude reduction in the liver parasite load. The protection achieved by MCSP + Vaxfectin or irradiated sporozoites differed significantly from any of the other CSP immunization groups (p<0.002) but did not differ from each other (p = 1.000).

**Figure 5:**
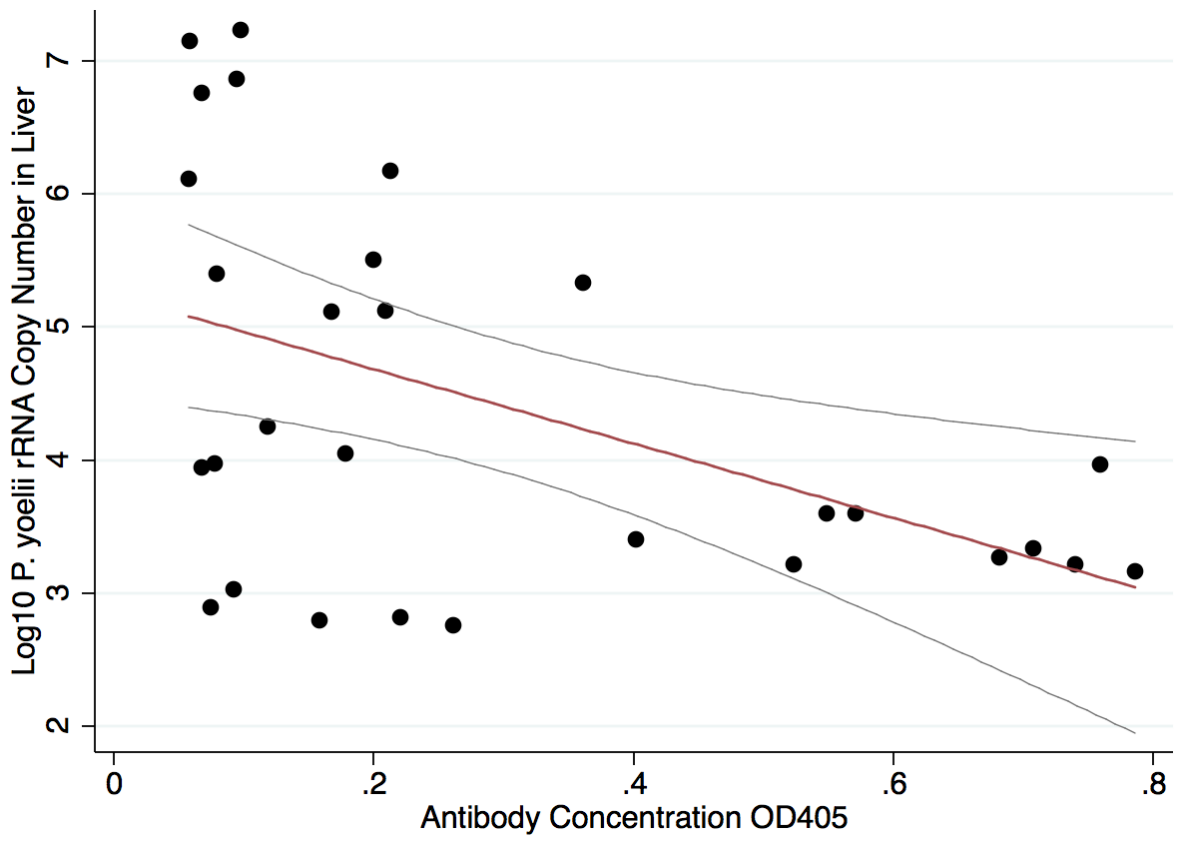
Association between anti-CSP antibody concentration and sporozoite infection of liver. Pearson correlation plot with regression line and 95% CI demonstrating the relationship between anti-CSP antibody concentration within individual mice and the log_10_ standardized copy number of *P. yoelii* rRNA recovered from the liver of the corresponding mouse, (r = 0.64, p<0.001).

We next examined the correlation between antibody concentrations in the sera of individual mice and the copies of rRNA that were detected in the liver sections. Figure 5 shows a scatter plot and regression line of the antibody concentrations and associated *P. yoelii* rRNA levels in the livers of immunized mice and demonstrates a strong correlation between the two measurements (r = .64, p = 0.0002).

### Role of T lymphocytes as effectors of MCSP-Vaxfectin elicited protective immunity

**Figure 6:**
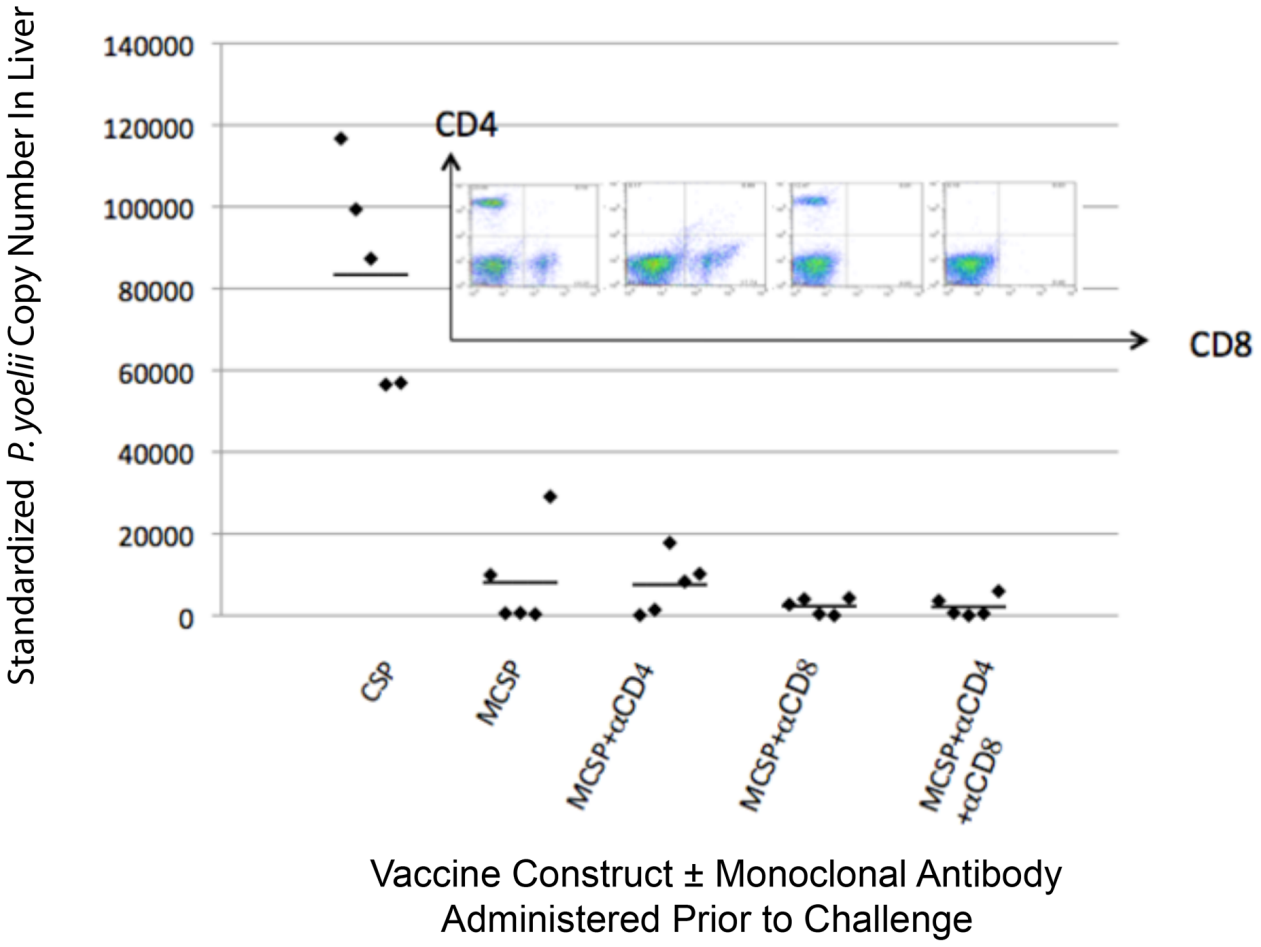
Effect of T cell depletion just prior to challenge on protection provided by Vaxfectin plus MCSP immunization. Two weeks after the final immunization, recipients of MCSP DNA were depleted of CD4, CD8 or both T cell subsets. The efficacy of the T-cell depletions is indicated by the flow cytometry analysis shown in the inset. The mice were then challenged with 2×10^3^ *P. yoelii* sporozoites. The standardized *P. yoelii* rRNA copy number in the livers of individual mice (5/group) and the mean for each group are shown. Not shown in the figure is that all immunized mice received Vaxfectin in addition to the indicated DNA constructs. The MCSP plus Vaxfectin immunized groups did not differ significantly from each other independent of T cell depletion (p = 1.000) and all MCSP + Vaxfectin immunized mice had significantly fewer rRNA copies than the CSP plus Vaxfectin immunized mice (p<0.001).

Because there is considerable evidence from irradiated sporozoite vaccine studies that T lymphocytes serve as important mediators of the elicited protective immunity at the pre-erythrocytic stage of infection [3]-[5], [8]-[10], [56]–[62], we sought to confirm further that in C57Bl/6 mice T cells were playing no role at the effector stage of the protective response elicited by our vaccine construct. This becomes important due to studies showing that C57Bl/6 mice are capable of generating a Class II-restricted effector T cell response [63]. Mice were immunized as previously described and two weeks after the final immunization were depleted of CD4+, CD8+ or both classes of T cells, as described in the Methods. A control group was immunized with Vaxfectin plus the CSP plasmid without the chemokine fusion component. The flow cytometric images in the inset of Figure 6 demonstrate that the respective monoclonal antibody treatments effectively eliminated the targeted T cell populations. Figure 6 also shows that the MCSP plus Vaxfectin immunized groups did not differ significantly from each other independent of T cell depletion (p = 1.000) and all MCSP + Vaxfectin immunized mice had significantly fewer rRNA copies in the liver than the CSP plus Vaxfectin immunized mice (p<0.001). These results support the conclusion that T lymphocytes play no role at the effector level in the observed protection.

### Characterization of the role of the adjuvant and the chemokine in eliciting enhanced responses

**Figure 7:**
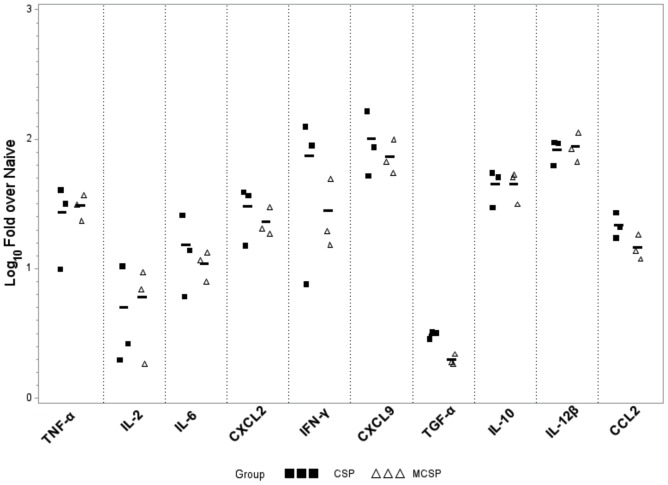
Expression of vaccine-induced cytokines and chemokines at immunization site 48 hours after immunization with and without MIP3α. Scatterplot showing results of an experiment in which 2 µg of Vaxfectin-formulated MCSP or Vaxfectin-formulated CSP were inoculated into the tibialis anterior muscle of C57BL/6 mice. The entire muscle was resected and processed as described in the Methods. Cytokine and chemokine levels of injected muscle samples were analyzed by real-time PCR 24 h (not shown) and 48 h after injection. There were no significant differences between the two time points. In all cases, the cytokine or chemokine levels did not differ between recipients of the CSP vs. the MCSP construct (p>0.5). All cytokine or chemokine levels for either DNA construct were significantly above control levels (p<0.002) except for levels of IL-2, and TGF-β, which did not differ significantly from control levels at either 24 or 48 hours (p>0.2).

The previous experiments demonstrate the marked enhancement of the immune response by use of the combination of Vaxfectin and the fusion of DNA encoding the chemokine MIP3α to that encoding CSP. To determine the role of these two vaccine components in eliciting the enhanced immune response, the relative ability of the Vaxfectin-CSP and Vaxfectin–MCSP regimens to elicit accumulation of reactive cells and enhanced cytokine and chemokine expression at the site of immunization 24 and 48 hours post-inoculation was evaluated. For this purpose, the entire tibialis major muscle at the immunization site was harvested from euthanized mice 24 and 48 hours post immunization with the adjuvant and the two constructs. Figure 7 demonstrates at 48 hours the expression at the site of immunization of a representative group of cytokines and chemokines associated with the initiation of an immune response. At both time points (24 hours not shown) the addition of MIP3α to the vaccine construct did not enhance the studied cytokine or chemokine levels over those observed with Vaxfectin and DNA encoding CSP alone (p>0.5).

We also examined the impact of the presence or absence of MIP3α in the vaccine construct on the cellular infiltrate that accumulated at the site of immunization. Table 1 shows that the combination of Vaxfectin and CSP DNA is the most potent attractant of cells to the site of inoculation. The recruitment of cells, and specifically dendritic cells, was not enhanced by inoculating with Vaxfectin and the fusion MIP3α-CSP DNA construct compared to Vaxfectin and CSP DNA without the MIP3α component (p = 1.000). The immunological enhancement observed with the combination of Vaxfectin and the MCSP construct therefore cannot be attributed to the chemoattractant functions of the chemokine. Further, the proportion of infiltrate contributed by CD11c+ cells was equivalent among all groups, other than that receiving only PBS, which differed significantly from the CSP/Vaxfectin group in the percentage of CD11c+ cells attracted.

**Table 1:** Impact of Different Immunization Regimens on Cellular Infiltrate at Site of Immunization.

	Total cell number (10^4^) (mean±SEM)	%CD11c+ cell(mean±SEM)	Total CD11c+ cell number (10^4^) (mean±SEM)
PBS	17.67±1.20	2.77±0.63	0.49±0.11
CSP	27.66±4.33	5.64±0.22	1.56±0.24
CSP/Vaxfectin	79.33±4.26*	6.46±0.97	5.05±0.52
MCSP	22.66±1.45	4.91±0.52	1.10±0.57
MCSP/Vaxfectin	83.66±8.57*	5.19±0.84	4.21±0.43

### Immune response to and protection provided by a vaccine construct targeting *P. falciparum*

**Figure 8:**
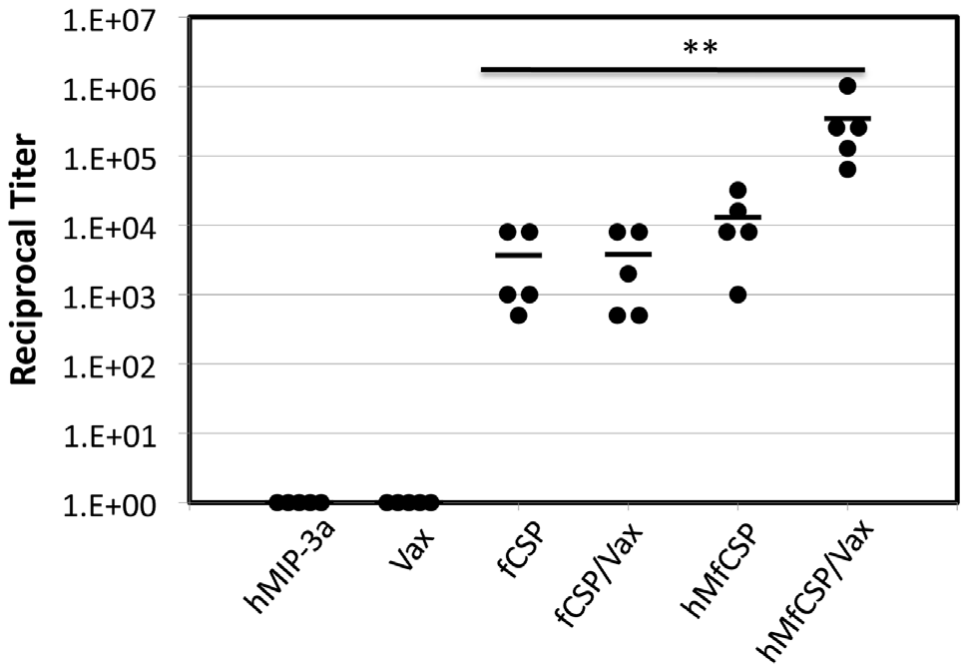
Antibody titers in mice immunized with the *P. falciparum* construct. C57BL/6 mice were immunized with 5 µg of the *P. falciparum* constructs of Figure 1 with or without Vaxfectin. Specific antibody concentrations. (Closed circles), which are reported as the highest dilution of serum at which the absorbance was twice the value obtained using pre-immunization serum, were determined two weeks after the final immunization. Also shown is the mean of the titers from the 5 mice in each group (lines). The response to the MIP3α-CSP/Vaxfectin immunization regimen was significantly greater than those to the other immunization regimens (p<0.01), as indicated by **.

A vaccine construct incorporating a portion of *P. falciparum* CSP DNA instead of *P. yoelii* CSP DNA was then developed and used to confirm the efficacy of this vaccine platform for reducing hepatic infection. This construct contained DNA encoding human MIP3α instead of murine MIP3α, both of which bind to murine CCR6. This vaccine construct would be one which would have potential clinical application by using the human MIP3α construct in a vaccine targeting the human malaria pathogen. In order to ensure adequate secretion of the MIP3α-fCSP protein complex, DNA encoding the tissue plasminogen activator signal sequence was incorporated into this vaccine construct. Mice immunized with the hMIP3α-CSP construct plus Vaxfectin had significantly higher antibody concentrations in their serum than did mice receiving any of the other Vaxfectin-containing combinations, including fCSP DNA plus Vaxfectin (p<0.01, Figure 8).

**Figure 9:**
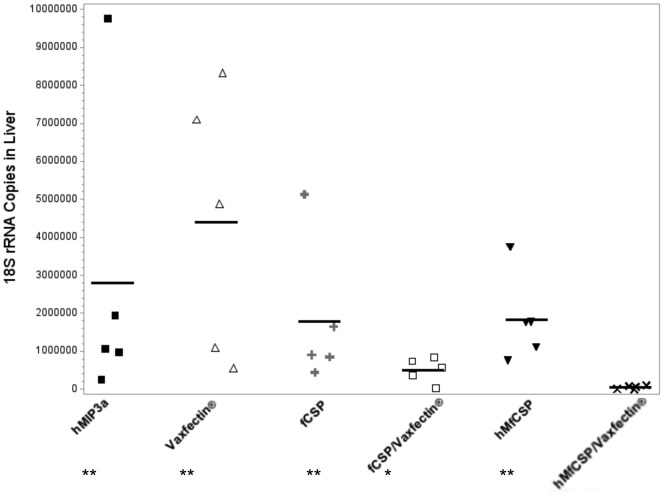
Protective efficacy of different vaccine constructs in C57BL/6 mice challenged with transgenic *P. berghei* expressing *P. falciparum* CSP. C57BL/6 mice were immunized with 5 µg of the indicated DNA vaccine as described in the Methods section. Two weeks after the final immunization, mice were challenged with 5×10^3^ transgenic *P.berghei* sporozoites, and parasite-specific rRNA levels in the liver were determined by quantitative RT-PCR on samples obtained 48 hours post challenge. All results were normalized against the expression of actin. Results indicate the copy numbers from individual mice (closed squares) and the mean from the 5 mice in each group (lines). ** Compared to hMfCSP/Vaxfectin p<0.001. *Compared to hMfCSP/Vaxfectin p<0.05. No other differences among groups were statistically significant.

Two weeks following the third immunization mice were challenged with 5×10^3^ transgenic *P. berghei* expressing CSP from *P. falciparum*. The hMIP3α-fCSP plus Vaxfectin protocol reduced the standardized quantity of *P. berghei* rRNA recovered from the liver by almost two orders of magnitude compared to the Vaxfectin alone control and one order of magnitude compared to the fCSP plus Vaxfectin immunization regimen (Figure 9, p<0.001 and p<0.05, respectively). In a separate experiment the ability of this vaccination protocol to protect against the development of blood-stage malaria was evaluated and compared to that in recipients of PBS, Vaxfectin plus a DNA construct encoding only hMIP3α without the fCSP component, and Vaxfectin and a DNA construct encoding CSP without hMIP3α. Only the hMfCSP plus Vaxfectin protocol provided significant protection in two independent challenge experiments (Table 2, Experiment 1 or 2, p = 0.05 vs. PBS). Although the same number of transgenic *P. berghei* sporozoites were used in both challenge experiments, sporozoites may vary in virulence among different harvests from the salivary gland of infected mosquitoes [43], as evidenced by the marked differences in outcomes among the groups receiving suboptimal vaccine regimens.

**Table 2:** Ability of different vaccine constructs to protect against the development of blood stage malaria infection.

Experiment 1	
*Immunization*	*No. Infected/No. Challenged*
PBS	4/5
hMIP3α/Vaxfectin	4/5
fCSP/Vaxfectin	2/5
hMfCSP/Vaxfectin	0/5

Experiment 2	
*Immunization*	*No. Infected/No. Challenged*
PBS	4/4
fCSP/Vaxfectin	5/5
hMfCSP	4/5
hMfCSP/Vaxfectin	1/5

To determine the actual concentration of specific antibody elicited by the different immunization protocols, pooled sera from mice used in the second experiment from Table 3 were assayed for antibody concentration by ELISA, using the fCSP-specific monoclonal antibody 2A10 as a reference standard. The results indicate a greater than 15-fold higher concentration of specific antibody in the group receiving the Vaxfectin-hM-fCSP regimen compared to the other immunization groups.

**Table 3:** CSP-specific Antibody Concentration in Pooled Sera from Different Groups of Immunized Mice.

Immunization Regimen	Antibody concentration (μg/ml)
fCSP/Vax	11.1
hMfCSP	105.3
hMfCSP/Vax	1638.9
PBS	0

## Discussion

The current studies demonstrate that a novel plasmid DNA-based immunization regimen can profoundly enhance the protective antibody response against malaria sporozoite challenge. The regimen used adjuvant to attract antigen-presenting cells to the site of immunization and, in addition, fused the vaccine antigen to a ligand that would specifically target it to immature dendritic cells. Use in a murine challenge model of the adjuvant Vaxfectin with a DNA construct encoding either the murine or human chemokine MIP3α used to CSP from *P. yoelii* or *P. falciparum* resulted in a greater than one log reduction in liver parasite burden compared to use of the construct without the chemokine and a reduction of greater than three orders of magnitude compared to that observed in CSP naïve mice. Where protection was compared to that achieved by immunization with irradiated sporozoites, the level of protection was equivalent. Further, the chemokine-CSP-adjuvant combination provided significantly enhanced protection against the development of blood stage malaria, a result not obtained with the regimen lacking the chemokine component.

MIP3α was included in the vaccine construct for the express purpose of targeting the vaccine antigen to the CCR6 receptor present on the immature dendritic cells that initiate an immune response. To determine the basis for the enhanced responses observed by use of the combination of adjuvant and chemokine, we examined the relative ability of the different compositions to attract cells involved in the immune response, particularly dendritic cells, to the site of immunization. These studies revealed that the combination of Vaxfectin and DNA encoding CSP was the most potent for attracting dendritic cells to the immunization site and that fusion of the chemokine encoding DNA to the CSP encoding DNA did not enhance the migration of those cells to the site. Although the chemotactic capability of MIP3α has been well documented [64]–[70], it is, in our studies, clearly less potent than Vaxfectin in attracting cells to the site of immunization. Further, the addition of the chemokine component to the vaccine construct did not alter the cytokine or chemokine profile within the inoculated tissue, compared to that observed with the use of Vaxfectin and vaccine encoding the CSP antigen alone. These observations would suggest that the role of the chemokine in this vaccine platform is simply to target the vaccine antigen to immature dendritic cells. In this vaccine platform, the traditional role of the adjuvant, attracting immune cells to the immunization site and creating an immunological milieu that favors the generation of adaptive immunity, is dependent on Vaxfectin. That MIP3α is acting solely as a targeting molecule is supported by previous studies indicating that actual fusion of the chemokine to the vaccine antigen was required for enhancement of immune responses observed with chemokine-antigen constructs without Vaxfectin [39].

Because of its demonstrated efficacy in the clinical setting, protection elicited by irradiated sporozoites has been the focus of much of the study of the immune mechanisms involved in protective immunity at the pre-erythrocytic stage of malaria infection [2], [3], [5], [13], [60], [71]–[77]. While previous studies have clearly demonstrated the protective potential of antibodies elicited by subunit vaccines [20], studies of vaccination with irradiated sporozoites have shown a critical role for CD8+ T cells in the elicited protection [3], [5], [8], [60], [78]–[81]. It has previously been demonstrated that C57Bl/6 mice fail to develop a Class I-restricted T cell response to *P. yoelii* CSP. The current studies further confirmed that neither CD4+ nor CD8+ T cells had any role at the effector stage in the observed protection.

The malaria vaccine we have described demonstrates a platform that can employ DNA to elicit highly protective humoral immune responses in mice. While some earlier studies have suggested poor immunogenicity of DNA vaccines in humans [82], more recent studies have indicated that use of adjuvants and alternative delivery systems have resulted in greatly enhanced immunogenicity in humans and/or non-human primates [83]–[90]. While further studies will be required to confirm the efficacy of this approach for sustained protection in non-murine vaccination settings, the protection observed with this vaccine platform demonstrates the potential for a DNA-based subunit vaccine to provide potent humoral immunity that prevented bloodstream infection after an intravenous challenge of C57Bl/6 mice with 1000 transgenic *P. berghei* sporozoites. Sedegah *et al* [43] studied protection using only Vaxfectin with a CSP DNA construct that lacked the MIP3α component and obtained maximum bloodstream protection of 54%, similar to what we observed with that construct, but clearly less than the 80–100% protection observed with the addition of the MIP3α component. In a more recent trial, Limbach *et al*. [91] could protect only 57% of mice challenged intravenously with 300 *P. yoelii* sporozoites after immunization with plasmid DNA followed by a vaccinia virus vector boost that expressed one, two or three different *P. yoelii* vaccine antigens. In one of the few studies to show a high level of protection, Scheiblhofer *et al.* demonstrated that DNA immunization with a CSP construct from which the glycosylphosphatidylinositol signal sequence was deleted elicited 100% protection of Balb/c mice from intradermal challenge with *P. berghei* [92]. In all of these studies, challenge occurred 14 days after the final immunization.

C57Bl/6 mice, in addition to being unable to generate a Class I-restricted T cell response to *P. yoelii* CSP, are also more susceptible to *P. berghei* infection than the Balb/c mice used by Scheiblhofer and others [24], [25]. Although intradermal or mosquito-based challenge may appear more representative of the clinical setting, it is more difficult to protect against direct intravenous challenge [93], [94]. Thus the potent antibody-based protection against bloodstream infection with transgenic *P. berghei* elicited by this subunit DNA vaccine was obtained under the most stringent challenge conditions.

Our study, like many others, demonstrates that DNA vaccines, properly formulated and delivered are capable of eliciting profound humoral immune responses. DNA vaccines also offer the advantages of rapid formulation and production, a long shelf life, an inability to revert into virulent forms, and ease of storage and transport, likely not requiring a cold chain, all issues highly relevant to the development of a malaria vaccine. However, the profound immune responses elicited by the platform described here supports its potential for application to other pathogens, as well.

## Supporting Information

Figure S1 **DNA sequence of pMCSP plasmid.** The IP-10 leader sequence: red; MIP3α: blue; CSP: black; Myc-Hisx6 tag: orange; Multiple cloning site/spacer: green. Translation start codon and stop codon are marked with boxes.
(TIF)

Figure S2 **DNA sequence of phMfCSP plasmid.** TPA leader sequence: red; hMIP3α: blue; fCSP: black; Myc tag: orange; Multiple cloning site/spacer: green. Translation start codon and stop codon are marked with boxes.
(TIF)
