# Supplementary figures and images for: Fusion of Antigen to a Dendritic Cell Targeting Chemokine Combined with Adjuvant Yields a Malaria DNA Vaccine with Enhanced Protective Capabilities
Source: PLoS One. 2014 Mar 5;9(3):e90413. doi: 10.1371/journal.pone.0090413 (PMC3943962; doi:10.1371/journal.pone.0090413)

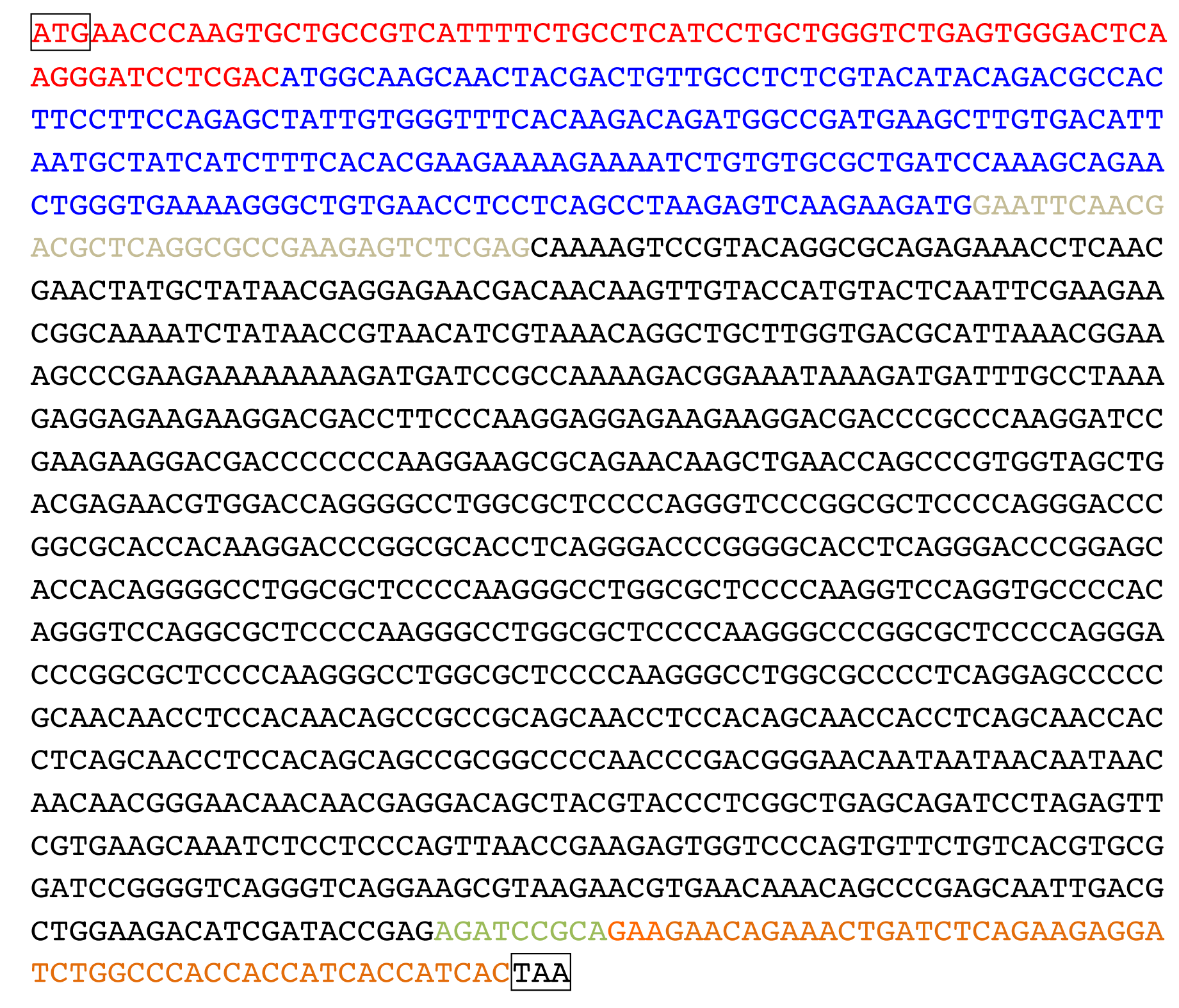

Supplement: Figure S1 — DNA sequence of pMCSP plasmid. The IP-10 leader sequence: red; MIP3α: blue; CSP: black; Myc-Hisx6 tag: orange; Multiple cloning site/spacer: green. Translation start codon and stop codon are marked with boxes. (TIF) [file pone.0090413.s001.tif]

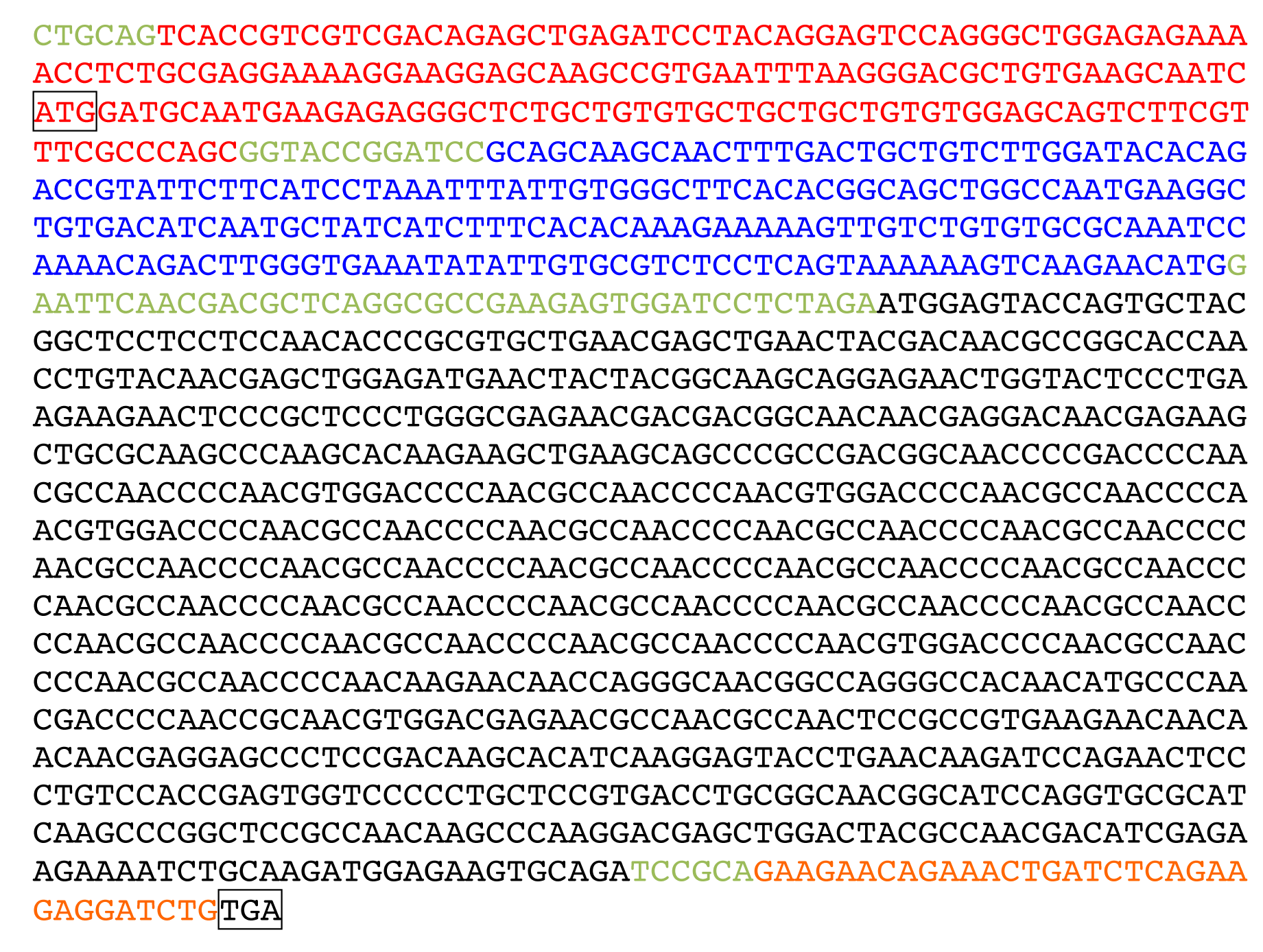

Supplement: Figure S2 — DNA sequence of phMfCSP plasmid. TPA leader sequence: red; hMIP3α: blue; fCSP: black; Myc tag: orange; Multiple cloning site/spacer: green. Translation start codon and stop codon are marked with boxes. (TIF) [file pone.0090413.s002.tif]
